# Identification of key genes and pathways associated with feed efficiency of native chickens based on transcriptome data via bioinformatics analysis

**DOI:** 10.1186/s12864-020-6713-y

**Published:** 2020-04-09

**Authors:** Lei Yang, Tingting He, Fengliang Xiong, Xianzhen Chen, Xinfeng Fan, Sihua Jin, Zhaoyu Geng

**Affiliations:** 1 College of Animal Science and Technology, Anhui Agricultural University, No. 130 Changjiang West Road, Hefei, 230036 China; 2 Key laboratory of local livestock and poultry genetic resource conservation and bio-breeding, Anhui Agricultural University, Hefei, 230036 People’s Republic of China

**Keywords:** Native chickens, RNA-seq, Residual feed intake, Feed efficiency, Transcriptome

## Abstract

**Background:**

Improving feed efficiency is one of the important breeding targets for poultry industry. The aim of current study was to investigate the breast muscle transcriptome data of native chickens divergent for feed efficiency. Residual feed intake (RFI) value was calculated for 1008 closely related chickens. The 5 most efficient (LRFI) and 5 least efficient (HRFI) birds were selected for further analysis. Transcriptomic data were generated from breast muscle collected post-slaughter.

**Results:**

The differently expressed genes (DEGs) analysis showed that 24 and 325 known genes were significantly up- and down-regulated in LRFI birds. An enrichment analysis of DEGs showed that the genes and pathways related to inflammatory response and immune response were up-regulated in HRFI chickens. Moreover, Gene Set Enrichment Analysis (GSEA) was also employed, which indicated that LRFI chickens increased expression of genes related to mitochondrial function. Furthermore, protein network interaction and function analyses revealed *ND2*, *ND4*, *CYTB*, *RAC2*, *VCAM1*, *CTSS* and *TLR4* were key genes for feed efficiency. And the ‘phagosome’, ‘cell adhesion molecules (CAMs)’, ‘citrate cycle (TCA cycle)’ and ‘oxidative phosphorylation’ were key pathways contributing to the difference in feed efficiency.

**Conclusions:**

In summary, a series of key genes and pathways were identified via bioinformatics analysis. These key genes may influence feed efficiency through deep involvement in ROS production and inflammatory response. Our results suggested that LRFI chickens may synthesize ATP more efficiently and control reactive oxygen species (ROS) production more strictly by enhancing the mitochondrial function in skeletal muscle compared with HRFI chickens. These findings provide some clues for understanding the molecular mechanism of feed efficiency in birds and will be a useful reference data for native chicken breeding.

## Background

Feed cost, account for 60–70% of the total cost of the modern poultry industry, has become one of the most important factors restricting the development of the poultry industry. A strategy to improve feed efficiency is a high priority for the poultry industry to reduce feed costs and nitrogen excretion [1]. Residual feed intake (RFI) has become a sensitive and accurate indicator of feed efficiency. RFI, first proposed by Koch et al. [2], is defined as the feed consumption above or below what is predicted for growth and maintenance. Herein, birds with low level RFI means consume less feed than predicted and are identified as efficient birds. For the last decades, RFI is being used to measure feed efficiency traits, which has successfully applied to the artificial selection of feed efficiency in mammal [3] and poultry [4]. Besides, RFI is a trait independent of other production traits, and the heritability of RFI is between 0.23 and 0.49 in chickens, these characteristics of RFI make it can be easily incorporated into the multi-trait selection indexes of commercial breeding companies [5]. From primary breeders to commercial growers, the selection of feed efficiency needs to be specifically considered by all industry practitioners to maximize returns. However, in fact, RFI is indeed rare in commercial production systems, mainly because of the complexity of RFI measurement [6]. In order to further expand the application prospect of RFI, it is urgent to study and explore the biological basis of chicken RFI.

RFI is a complex quantitative trait that is known to be associated with many biological factors. High throughput sequencing technology including RNA sequencing (RNA-seq) has become a mature and efficient tool for deeper understanding the underlying molecular mechanism of complex trait such as RFI [7]. An earlier duodenal transcriptomic analysis in chickens showed that the difference in RFI may be related to digestibility, metabolism and biosynthesis processes as well as energy homeostasis [8]. Moreover, A previous high throughput sequencing analysis indicates that mitochondrial energy metabolism in skeletal muscle plays a key role in the regulation of feed efficiency [9, 10]. Skeletal muscle plays a particularly important role in the utilization and storage of carbohydrates and lipids from feed [11]. In chickens, the breast muscle is the main skeletal muscle. Many studies have reported that feed efficiency is associated with mitochondria function, breast muscle growth, and some physiological changes of breast muscle tissue in chickens [10, 12, 13].

Statistically, RNA-seq has been widely used for in-deep analysis and a better understanding of the molecular basis of feed efficiency in chickens. To further interpret RNA-seq data, functional enrichment analysis is extensively used to derive biological insight. Traditionally, differentially expressed genes (DEGs) from transcriptome data were first identified, and then the biological interpretation of DEGs was assisted by computational functional analysis based on accumulated biological knowledge. Finally, the biological function analysis of DEGs is based on a list of preselected ‘interesting’ genes [14]. However, traditional practices in transcriptomic data analysis can only account for DEGs selected by arbitrary cutoffs, and this method may also be limiting insight by prioritizing highly differentially expressed and ‘interesting’ genes over those genes that undergo moderate fold-changes [15]. Gene Set Enrichment Analysis (GSEA) is a computational method used to determine whether a particular gene expression pattern is significantly different between two groups of samples [16]. GSEA is reviewed as a cutoff-free strategy, which ranks all expressed genes according to the strength of expression difference. Compared with biological function analysis of DEGs, GSEA method avoids choosing arbitrary cutoffs and can accumulate subtle expression changes in the same group of gene set for studying functional enrichment between two biological groups [17]. In the current study, transcriptome data were analyzed with DEGs function analysis and GSEA method in order to obtain comprehensive biological insight of differences between RFI groups.

Wannan Yellow chicken was selected as experiment material. It is a famous native chicken breed and popular in the southeast of China for its delicious meat and unique flavor. There is considerable variation in feed efficiency between commercial broilers and native chickens. In addition to extrinsic factors such as diet, microbiota, and housing environment, it can be speculated that there are some internal molecular mechanism enabling the differential allocation of resources for various physiological processes [18]. The transcriptome data from commercial broilers may not be appropriate as a reference for native chicken breeding. To date, however, a large number of sequencing analyses have been performed on commercial broilers [12, 19], but only a few reports have focused on native chickens [20]. Therefore, the objective of this study was to identify a series of key genes and pathways affecting feed efficiency through analysis of the breast muscle transcriptome between native chickens divergent with extreme RFI. Our findings will contribute to a better understanding of the underlying molecular mechanism of feed efficiency and provide important reference information for native chicken breeding.

## Results

### Performance and feed efficiency

The difference in feed intake, growth performance, and feed efficiency traits are showed in Table 1. The average daily feed intake (ADFI) of HRFI birds was significantly higher than that of LRFI birds (*P* < 0.05), and the HRFI group consumed 8.8% more feed than the LRFI group. As expected, the FCR and RFI of LRFI group were significantly lower than those of HRFI group (*P* < 0.05). the LRFI birds had the RFI value of − 2.29 ± 0.16 compared with 1.94 ± 0.09 for the HRFI birds during 42 days (day 56–98) of the experiment. In addition, there was no significant difference in the initial body weight (BW56) and final body weight (BW98) between RFI groups (*P* > 0.05). Moreover, metabolic body weight (MBW^0.75^) and average daily body weight gain (ADG) also showed no significant difference between the two groups (*P* > 0.05).

**Table 1:** Characterization of performance and feed efficiency traits (Least square means and SEM)

Traits^a^	HRFI group, *n* = 30	LRFI group, *n* = 30	*P*-value
BW56, g	460.70 ± 6.54	460.40 ± 4.06	0.813
BW98, g	956.08 ± 15.91	990.36 ± 10.48	0.071
ADFI, g/d	41.55 ± 0.59	38.19 ± 0.50	< 0.001
ADG, g/d	11.82 ± 0.32	12.56 ± 0.17	0.058
MBW^0.75^, g	137.56 ± 1.38	140.00 ± 1.03	0.143
FCR, g/g	3.71 ± 0.07	2.99 ± 0.02	< 0.001
RFI, g	1.94 ± 0.09	−2.29 ± 0.16	< 0.001

^a^ *BW56* initial body weight, *BW98* final body weight, *ADFI* average daily feed intake, *ADG* average daily body weight gain, *MBW*^*0*.*75*^ metabolic body weight, *FCR* feed conversion ratio, *RFI* residual feed intake

### Gene expression profile

**Fig. 1:**
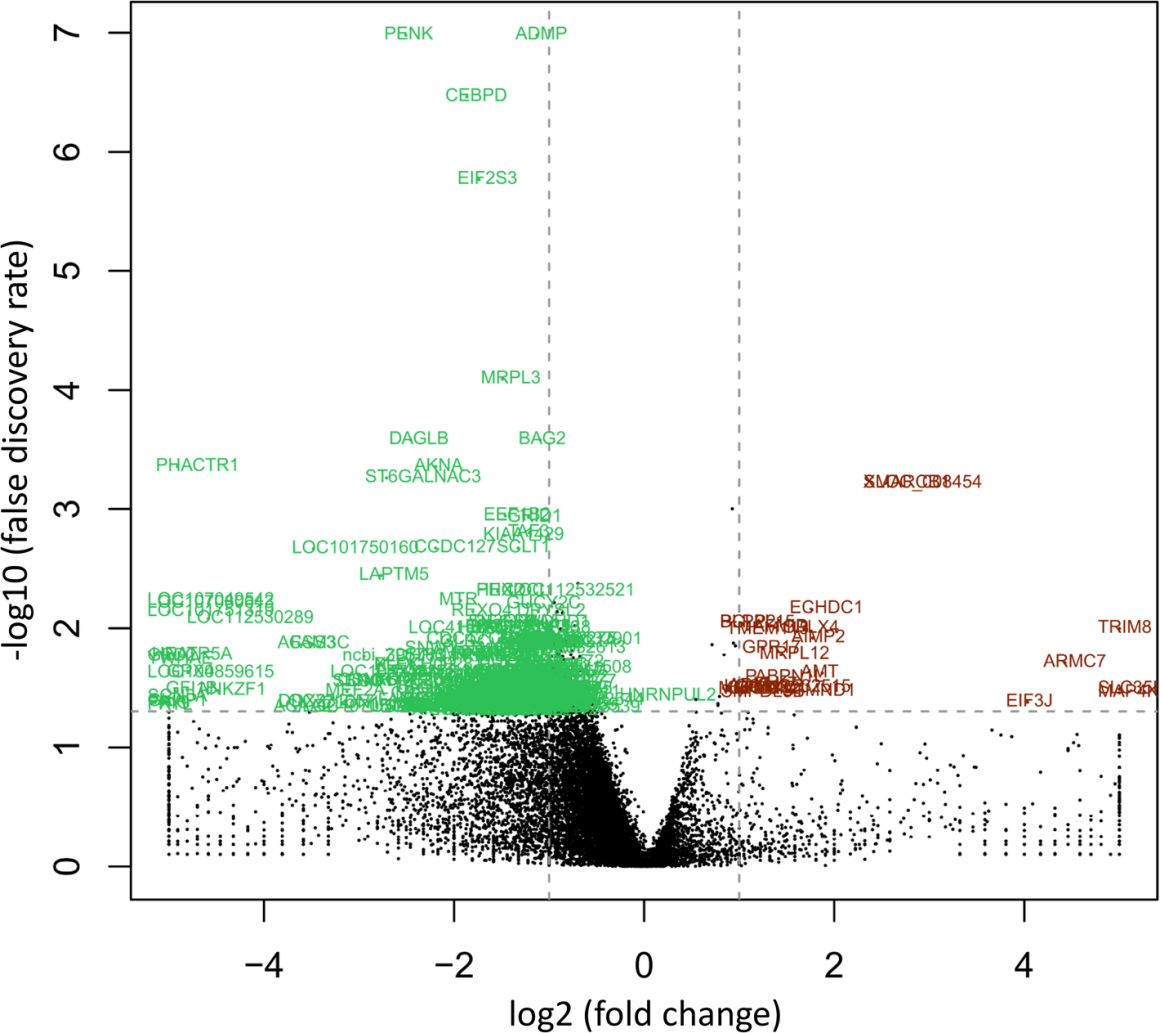
Volcano plot of differently expressed genes (DEGs). The volcano plots illustrate the size and significance of the differentially expressed genes (DEGs) between HRFI and LRFI groups. Red dots are up-regulated genes and green dots are down-regulated genes in LRFI chickens

All breast muscle samples (*n* = 5 per RFI group) were collected for RNA-seq. The number of raw reads, high quality raw reads, trimmed reads, and mapped reads for each sample are presented in (Additional file 1: Table S1). After filter, the overall Q30 percentage of high quality clean data was above 95%. An average of 68.1 million trimmed reads per sample were mapped to the reference with a mean of 83.05% mapping efficiency. To analyze the transcriptional variations occurring between the HRFI and LRFI groups, differential gene expression analysis was used in the current study. Among all the genes annotated in the chicken genome, after multiple tests and corrections, a total of 354 gens were identified as being DEGs (Fig. 1). 5 DEGs were detected within the unannotated parts of the chicken genome, which could be considered as novel genes. Of the 349 known DEGs, 24 DEGs were up-regulated in the LRFI groups while 325 were down-regulated compared with the HRFI groups. Of the up-regulated known genes, 19 DEGs had a fold change between 2 and 4, and 5 DEGs had a fold change > 4. Of the down-regulated known genes, 263 DEGs had a fold change between − 2 and − 4, and 62 DEGs had a fold change < − 4. The list of the top 10 known up- and down-regulated DEGs in the breast muscle of LRFI group, ranked by log2 (fold change) (log2FC), are showed in Table 2. The most altered genes in LRFI group were *C24H11orf34* (up-regulated, log2FC = 10.09, false discovery rate (FDR) = 0.033) and *RHNO1* (down-regulated, log2FC = − 7.57, FDR = 0.017). Moreover, a complete list of DEGs is presented in (Additional file 2: Table S2).

**Table 2:** Top 10 known up- and down-regulated differently expressed genes (DEGs) in LRFI group

Gene symbol	Log2FC	*P*-value	FDR^a^	Description	HRFI vs LRFI
*C24H11orf34*	10.09	5.26E-04	0.033	Chromosome 24 C11orf34 homolog	up
*FCGBP*	5.77	2.85E-05	0.010	Fc fragment of IgG binding protein	up
*GUCA2B*	5.27	7.40E-04	0.035	Guanylate cyclase activator 2B	up
*MUC2*	4.43	1.59E-04	0.019	Mucin 2, oligomeric ucus/gel-forming	up
*CDHR2*	4.03	1.16E-03	0.042	Cadherin related family member 2	up
*BFSP1*	1.87	2.45E-04	0.024	Beaded filament structural protein 1	up
*ND2*	1.78	4.23E-05	0.012	NADH dehydrogenase subunit 2	up
*CYTB*	1.76	1.22E-05	0.007	Cytochrome b	up
*ND4*	1.68	2.95E-05	0.010	NADH dehydrogenase subunit 4	up
*LOC101748207*	1.68	7.03E-04	0.034	Soluble scavenger receptor cysteine-rich domain-containing protein SSC5D-like	up
*AICDA*	−5.05	1.44E-04	0.018	Activation induced cytidine deaminase	down
*LOC107049096*	−5.09	1.42E-05	0.007	GTPase IMAP family member 8-like	down
*TLX2*	−5.25	2.43E-04	0.024	T-cell leukemia homeobox 2	down
*LOC112531272*	−5.43	1.02E-05	0.006	Osteoclast-associated immunoglobulin-like receptor	down
*LOC107050476*	−5.83	8.96E-06	0.006	Uncharacterized LOC107050476	down
*TMEM150B*	−6.27	1.41E-03	0.045	Transmembrane protein 150B	down
*LECT2*	−6.64	9.11E-04	0.038	Leukocyte cell derived chemotaxin 2	down
*LOC429329*	−6.88	1.11E-03	0.041	Solute carrier family 30 member 2	down
*SLC30A2*	−6.88	1.27E-03	0.043	T-cell-interacting, activating receptor on myeloid cells protein 1-like	down
*RHNO1*	−7.57	1.29E-04	0.017	RAD9-HUS1-RAD1 interacting nuclear orphan 1	down

^a^ *FDR* false discovery rate

### Functional enrichment of DEGs

A functional enrichment analysis was performed to reveal the potential functional categories of DEGs. Analysis of Gene Ontology (GO) enrichment for the DEGs indicated that 212 biological processes terms were significantly enriched, which were mainly associated with ‘immune system processes’ and ‘response to stimulus’. Moreover, the significantly enriched GO terms also including 17 cellular component terms and 12 molecular function terms, which involved in ‘plasma membrane part’ and ‘carbohydrate derivative binding’. All enriched GO terms of DEGs are provided in (Additional file 3: Table S3).

**Fig. 2:**
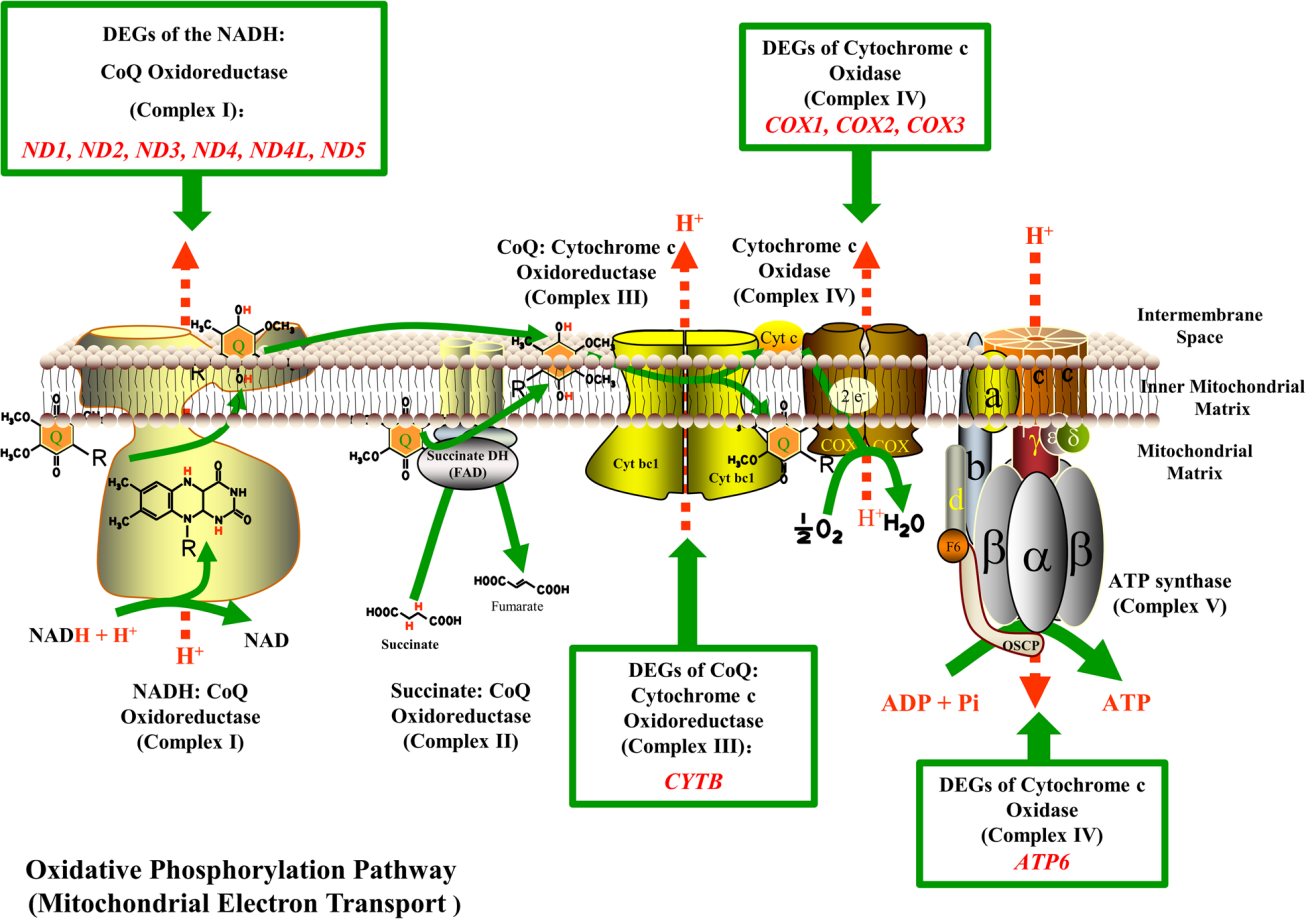
Oxidative phosphorylation signaling enriched of differentially expressed genes (DEGs). The DEGs of oxidative phosphorylation signaling were mainly enriched in complex I, complex III, complex IV, and complex V. The scheme shows oxidative phosphorylation signaling and was visualized by ScienceSlides tool (http://www.visiscience.com/scienceslides). The DEGs of oxidative phosphorylation signaling are shown in the green box, and the gene symbol in red indicates that the gene is up-regulated in the LRFI group

Enrichment of the Kyoto Encyclopedia of Genes and Genomes (KEGG) pathways was performed to further reveal the biological functions of DEGs [21]. Interestingly, genes of ‘oxidative phosphorylation’ were up-regulated in LRFI group (Fig. 2), while genes of other enriched pathways were down-regulated in LRFI group (Table 3). Other enriched pathways of interest including ‘cytokine-cytokine receptor interaction’ and ‘Jak-STAT signaling pathway’, which were involved in muscle myogenesis and regulation of immune response. The remaining significant enriched signaling pathways, such as ‘phagosome’, ‘cell adhesion molecules (CAMs)’, and ‘toll-like receptor signaling’, were mainly involved in inflammation, immune response, and innate immune response.

**Table 3:** All enriched KEGG pathway-based sets of differentially expressed genes (DEGs) in between RFI groups

Signaling pathways	Count	B-H *P-*value	Genes^a^
Phagosome	17	0.0001	*TLR4, TUBB6, BF2, NCF4, BLB1, CYBB, TLR2B, THBS1, BLB2, ACTB, CTSS, ITGB2, DMB2, TAP1, TAP2, LOC100859737, YF5*
Cell adhesion molecules (CAMs)	15	0.0003	*BF2, BLB1, ICOS, BLB2, CD8BP, ITGA8, ITGB2, PTPRC, NLGN1, DMB2, YF5, ITGA4, VCAM1, PDCD1LG2*
Intestinal immune network for IgA production	8	0.0003	*BLB1, ICOS, AICDA, BLB2, TNFSF13B, DMB2, ITGA4*
Cytokine-cytokine receptor interaction	18	0.0003	*TNFRSF18, FASLG, XCR1, EDA2R, IL18R1, CSF2RA, TNFSF13B, CCL1, CCR2, IL4R, TNFRSF8, IL18, TNFRSF4, IL17RA, IL22RA2, IL1RAP, TNFRSF25, TNFSF4*
Oxidative phosphorylation	11	0.0065	***ND1, ND2, ND3, ND4, ND4L, ND5, CYTB, COX1, COX2, COX3, ATP6***
Toll-like receptor signaling pathway	9	0.0140	*TLR4, TLR2B, SPP1, TRAF3, PIK3CB, STAT1, PIK3R5, PIK3CD, TLR1B*
Jak-STAT signaling pathway	11	0.0353	*CSF2RA, SOCS3, JAK3, PIM1, PIK3CB, STAT1, IL4R, PIK3R5, PIK3CD, IL22RA2, PTPN6*
Regulation of actin cytoskeleton	13	0.0412	*TMSB4X, ARPC5, RAC2, ITGA8, ACTB, PIK3CB, IQGAP2, ITGB2, ARPC1B, PIK3R5, PIK3CD, CYFIP2, ITGA4*

^a^ Up-regulated genes in LRFI birds are highlighted in bold and down-regulated genes in normal typeface

### Identification of hub genes and pathways through protein-protein interaction (PPI) network analysis of DEG

**Fig. 3:**
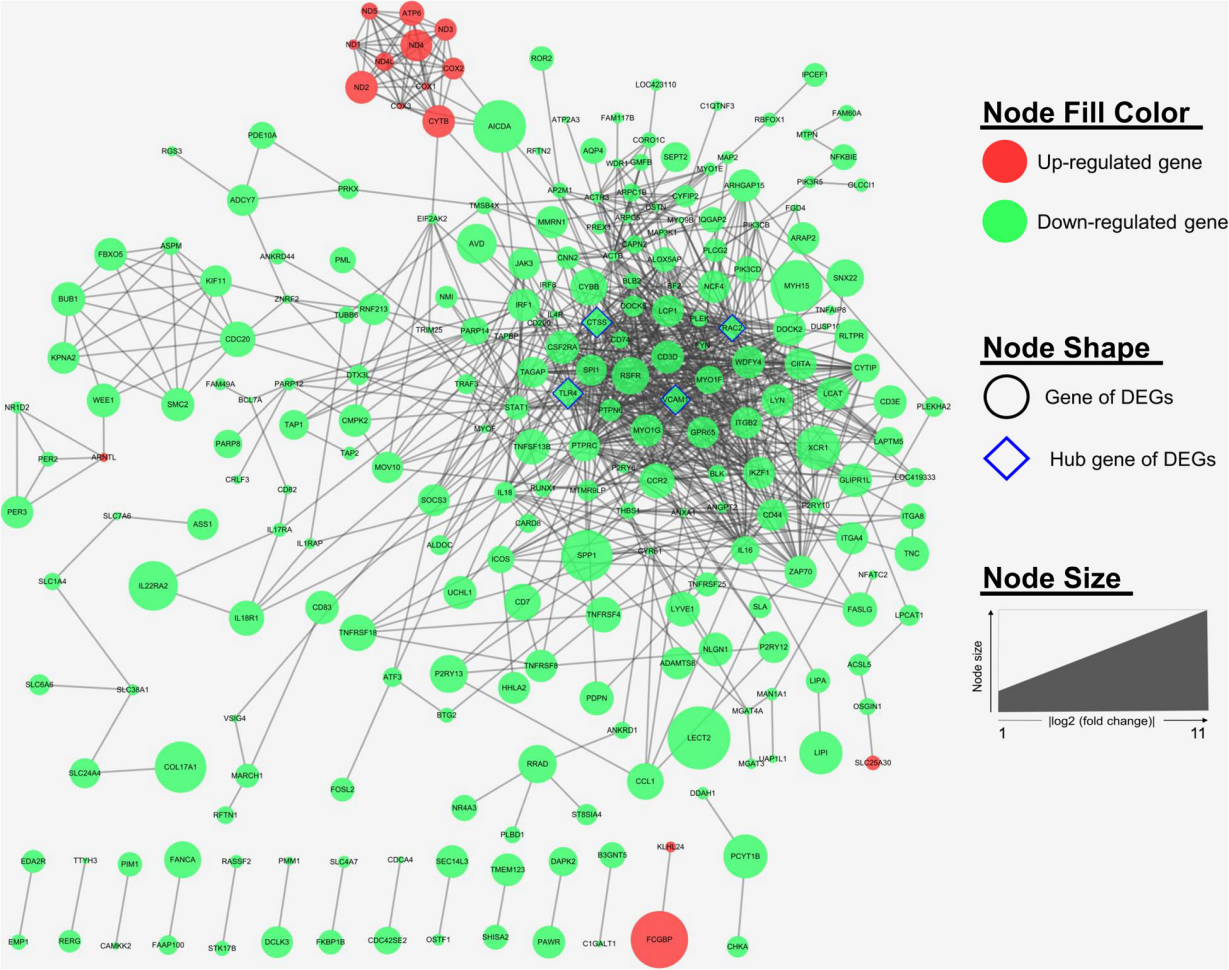
Protein-protein interaction (PPI) network for products of differentially expressed genes (DEGs). A total of 245 nodes and 942 interaction associations were identified. The red node represents the up-regulated gene, while the green node represents the down-regulated gene. The nodes with the highest degree scores were shaped as diamond and highlight the blue border paint. Node size indicated the fold change of each gene

**Fig. 4:**
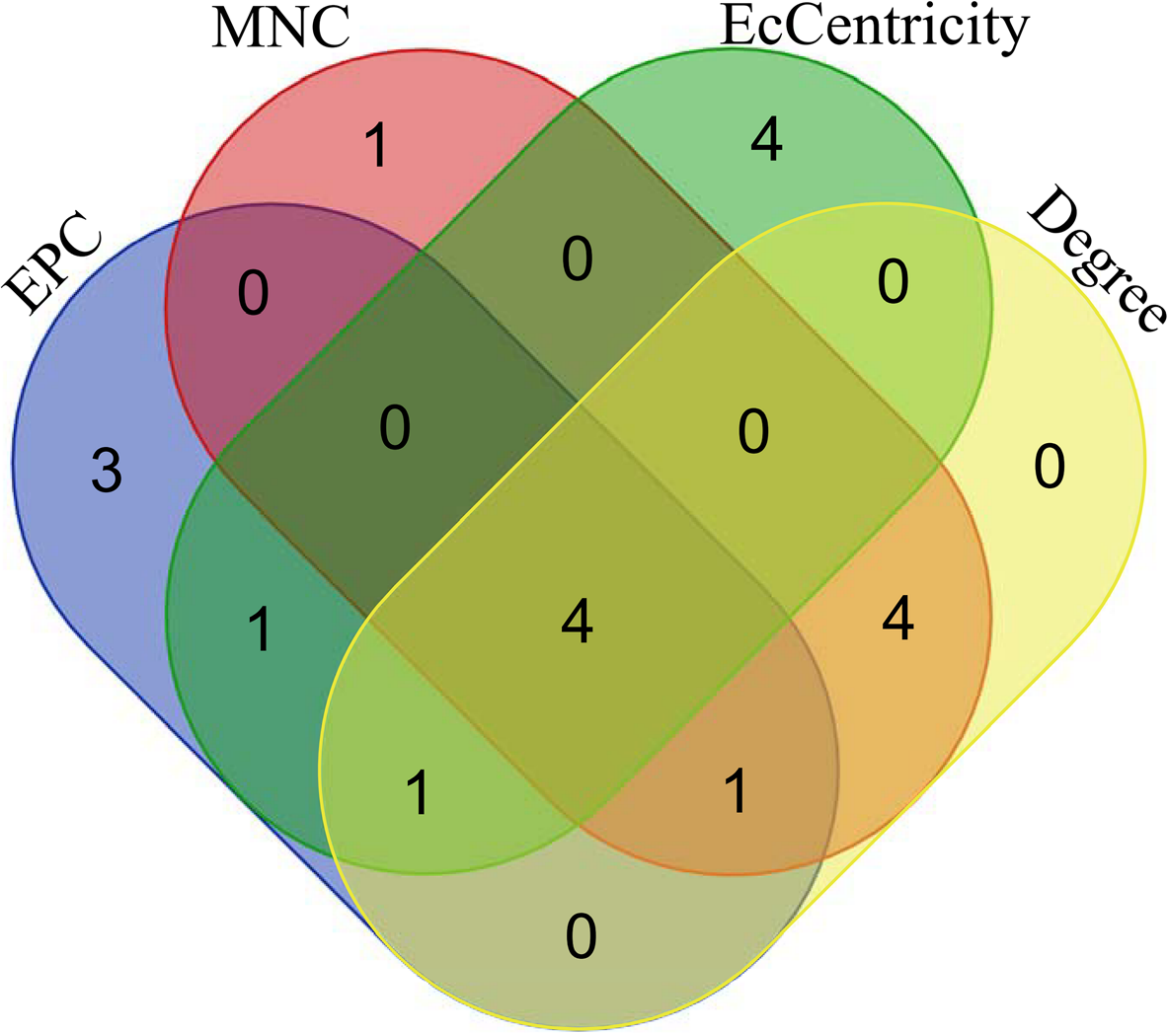
Venn plot to identify significant hub genes generated by four centrality methods. The four methods were Degree, EPC, EcCentricity, and MNC. Areas with different colors correspond to different algorithms. The cross areas indicate the commonly accumulated differentially expressed genes (DEGs). The elements in concurrent areas are the 4 hub genes (*RAC2*, *VCAM1*, *CTSS*, and *TLR4*)

The PPI network analysis was employed to further analyze and reveal the interaction information of DEGs. The PPI network of DEGs, including 245 nodes and 942 edge, was constructed in the STRING database and visualized using Cytoscape software (Fig. 3). The cutoff criterion was set as degree > 10. Base on the STRING database, the top 10 genes of DEGs evaluated in the PPI network using four centrality methods (Table 4). Moreover, we observed the intersections of these four algorithms and generated a Venn plot (Fig. 4) to identify significant hub genes using an online website (http://bioinformatics.psb.ugent.be/webtools/Venn/). Finally, the four hub genes, including *RAC2* (Ras-related C3 botulinum toxin substrate 2), *VCAM1* (Vascular cell adhesion molecule 1), *CTSS* (Cathepsin S), and *TLR4* (Toll like receptor 4), were identified. Among these genes, *RAC2* showed the highest node degree, which was 50. The hub genes derived using these four algorithms may represent key candidate genes with important biological regulatory functions.

**Table 4:** Top 10 genes evaluated in the protein-protein interaction (PPI) network

Gene	Degree	Gene	EPC	Gene	EcCentricity	Gene	MNC
*PTPRC*	56	*IL16*	134.471	*TLR4*	0.141497	*PTPRC*	56
*RAC2*	50	*TLR4*	134.471	*STAT1*	0.141497	*RAC2*	50
*MYO1F*	42	*PTPN6*	134.471	*PTPN6*	0.141497	*MYO1F*	42
*ITGB2*	39	*CTSS*	134.471	*CTSS*	0.141497	*SPI1*	39
*SPI1*	39	*RAC2*	134.471	*RAC2*	0.141497	*ITGB2*	38
*VCAM1*	38	*VCAM1*	134.471	*VCAM1*	0.141497	*CTSS*	37
*CTSS*	37	*ITGB2*	134.471	*ACTB*	0.141497	*VCAM1*	37
*ACTB*	36	*ACTB*	134.471	*TAGAP*	0.141497	*IKZF1*	35
*TLR4*	35	*CD3D*	134.471	*FYN*	0.141497	*TLR4*	34
*IKZF1*	35	*GPR65*	134.471	*LYN*	0.141497	*MYO1G*	33

**Fig. 5:**
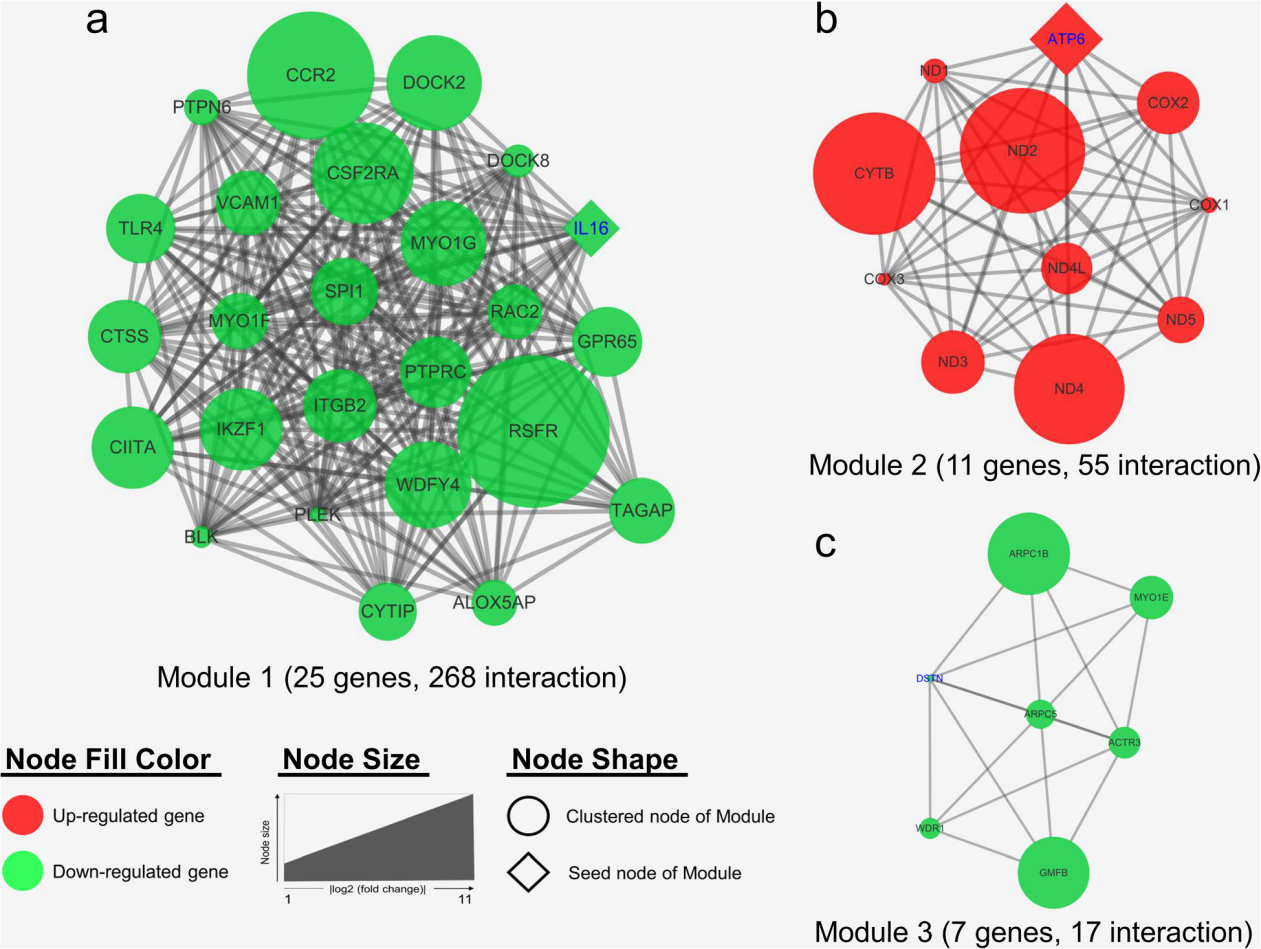
The three protein-protein interaction (PPI) hub network modules. The three significant modules, including (**a**) module 1 (MCODE score = 22.33), **b** module 2 (MCODE score = 11), and **c** module 3 (MCODE score = 5.67), were constructed from PPI network of DEGs using MCODE. The red node represents the up-regulated gene, while the green node represents the down-regulated gene. The seed node of each module was shaped as diamond and highlight the blue gene symbol. Node size indicated the fold change of each gene

The three significant modules, including module 1 (MCODE score = 22.33), module 2 (MCODE score = 11), and module 3 (MCODE score = 5.67), were constructed from the PPI network of the DEGs by MCODE (Fig. 5). And then, genes of each module were performed by biological functional enrichment analysis, respectively (Table 5). Module 1 (Fig. 5a), including 25 nodes and 268 edges, were significantly enriched in ‘immune system process’, ‘phagosome’, and ‘cell adhesion molecules (CAMs)’. Moreover, module 2 (Fig. 5b), including 11 nodes and 55 edges, were markedly enriched in ‘ATP synthesis coupled electron transport’, ‘ATP metabolic process’, and ‘oxidative phosphorylation’. Furthermore, module 3 (Fig. 5c) contains 7 nodes and 17 edges that are mainly involved in ‘regulation of actin filament length’, ‘salmonella infection’, and ‘regulation of actin cytoskeleton’. In addition, a complete result of enrichment analysis of genes in each module are shown in (Additional file 4: Table S4).

**Table 5:** The biological function enrichment analysis of the three protein-protein interaction (PPI) hub network modules

Module	Database	GO term / pathway	B-H *P-*value	Genes^a^
Module 1	GO	Cell activation	3.13E-06	*PTPRC, SPI1, IKZF1, RAC2, DOCK2, PTPN6, PLEK, DOCK8, TLR4, ITGB2*
		Immune system process	5.23E-06	*PTPRC, SPI1, IKZF1, BLK, RAC2, CCR2, DOCK2, PTPN6, PLEK, IL16, DOCK8, TLR4, ITGB2*
		Leukocyte activation	7.68E-06	*PTPRC, SPI1, IKZF1, RAC2, DOCK2, PTPN6, DOCK8, TLR4, ITGB2*
	KEGG	Phagosome	0.024684	*CTSS, TLR4, ITGB2*
		Cell adhesion molecules (CAMs)	0.024684	*PTPRC, VCAM1, ITGB2*
Module 2	GO	Cellular respiration	1.35E-09	***ND4L, ND4, ND5, ND1, COX3, CYTB***
		ATP synthesis coupled electron transport	1.63E-09	***ND4L, ND4, ND5, COX3, CYTB***
		ATP metabolic process	2.70E-09	***ATP6, ND4L, ND4, ND5, COX3, CYTB***
	KEGG	Oxidative phosphorylation	1.85E-08	***ATP6, ND3, ND4L, ND4, ND1***
		Metabolic pathways	0.001906	***ATP6, ND3, ND4L, ND4, ND1***
Module 3	GO	Regulation of actin filament length	7.35E-10	*DSTN, WDR1, ARPC5, GMFB, ARPC1B, ACTR3*
		Regulation of actin polymerization or depolymerization	7.35E-10	*DSTN, WDR1, ARPC5, GMFB, ARPC1B, ACTR3*
		Actin polymerization or depolymerization	9.09E-10	*DSTN, WDR1, ARPC5, GMFB, ARPC1B, ACTR3*
	KEGG	Salmonella infection	0.002892	*ARPC5*, *ARPC1B*
		Regulation of actin cytoskeleton	0.010779	*ARPC5*, *ARPC1B*

^a^ Up-regulated genes in LRFI birds are highlighted in bold and down-regulated genes in normal typeface

### GSEA

**Fig. 6:**
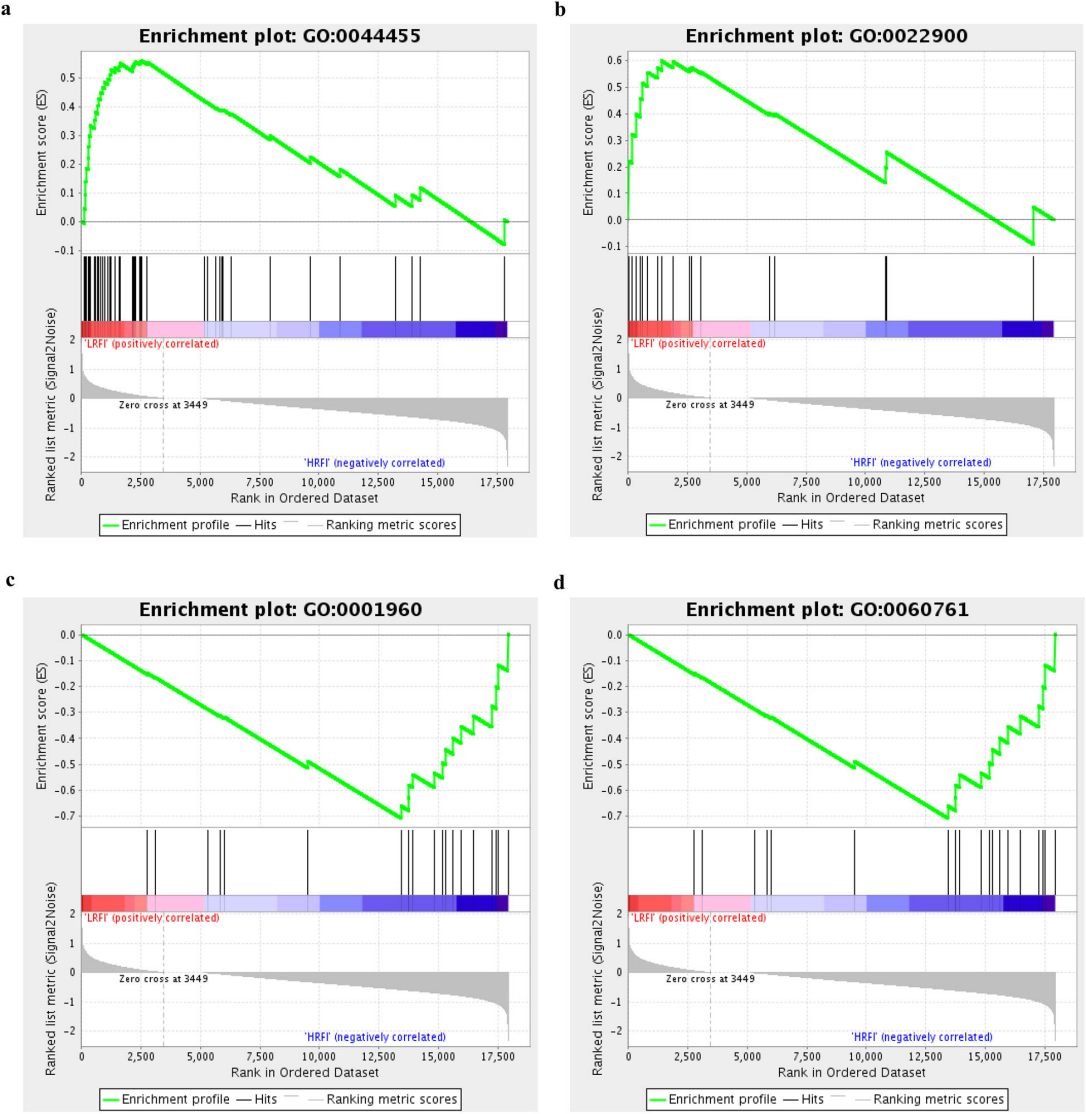
Gene set enrichment analysis (GSEA). GSEA was performed in the HRFI and LRFI groups. The GSEA algorithm calculates an enrichment score reflecting the degree of overrepresentation at the top or bottom of the ranked list of the genes included in a gene set in a ranked list of all genes present in the RNA-seq dataset. A positive enrichment score (ES) indicates gene set enrichment at the top of the ranked list; a negative ES indicates gene set enrichment at the bottom of the ranked list. The analysis demonstrates that known (**a**) Mitochondrial membrane part and (**b**) Electron transport chain, are enriched in LRFI groups, while (**c**) Negative regulation of cytokine-mediated signaling pathway and (**d**) Negative regulation of response to cytokine stimulus are enriched in HRFI groups

We further investigated the difference of gene expression levels between HRFI and LRFI groups by GSEA. GSEA was performed using a GO-based list, including 9996 gene sets, and a KEGG-based list, including 186 gene sets. Moreover, the results of GSEA analysis are presented in Additional file 5: Table S5. Totally, 20 gene sets, including 14 GO-based gene sets and 6 KEGG-based gene sets, were identified as significantly enriched (Table 6) (FDR < 0.05). Positive and negative NES indicate higher and lower expression in LRFI, respectively. From the GO-based list, interestingly, all higher expression gene sets in LRFI group were mainly related to mitochondrial function, such as ‘mitochondrial membrane part’ (Fig. 6a) and ‘electron transport chain’ (Fig. 6b). On the other hand, the lower expression gene sets in LRFI group were involved in inflammatory response, response to stimulus, molecular transport, and metabolic process, such as ‘negative regulation of cytokine-mediated signaling pathway’ (Fig. 6c) and ‘negative regulation of response to cytokine stimulus’ (Fig. 6d). From the KEGG-based list, the higher expression gene sets in LRFI group were ‘citrate cycle (TCA cycle)’ and ‘cardiac muscle contraction’. And the higher expression gene sets in HRFI group were ‘intestinal immune network for IgA production’, ‘N-Glycan biosynthesis’, ‘apoptosis’, and ‘glycosaminoglycan biosynthesis-chondroitin sulfate/dermatan sulfate’.

**Table 6:** Gene set enrichment analysis (GSEA) between HRFI and LRFI birds

Gene set		NES^a^	FDR^b^	Higher expression in HRFI or LRFI
GO-based list (C5, CC, C5.BP, C5.MP)				
GO:0044455	Mitochondrial membrane part	2.60	< 0.001	LRFI
GO:0022900	Electron transport chain	2.09	0.011	LRFI
GO:0010822	Positive regulation of mitochondrion organization	1.80	0.027	LRFI
GO:0005740	Mitochondrial envelope	1.82	0.029	LRFI
GO:0009205	Purine ribonucleoside triphosphate metabolic process	1.85	0.031	LRFI
GO:0009144	Purine nucleoside triphosphate metabolic process	1.91	0.034	LRFI
GO:0046034	ATP metabolic process	1.85	0.039	LRFI
GO:0001960	Negative regulation of cytokine-mediated signaling pathway	−1.77	0.010	HRFI
GO:0060761	Negative regulation of response to cytokine stimulus	−1.76	0.011	HRFI
GO:0070588	Calcium ion transmembrane transport	−1.74	0.012	HRFI
GO:1903169	Regulation of calcium ion transmembrane transport	− 1.73	0.015	HRFI
GO:0042439	Ethanolamine-containing compound metabolic process	−1.71	0.019	HRFI
GO:0001776	Leukocyte homeostasis	−1.70	0.022	HRFI
GO:0008625	Extrinsic apoptotic signaling pathway via death domain receptors	−1.70	0.023	HRFI
KEGG-based list (C2.CP:KEGG)				
KO00020	Citrate cycle (TCA cycle)	2.28	0.005	LRFI
KO04260	Cardiac muscle contraction	1.70	0.031	LRFI
KO04672	Intestinal immune network for IgA production	−1.60	0.020	HRFI
KO00510	N-Glycan biosynthesis	−1.57	0.022	HRFI
KO04210	Apoptosis	−1.57	0.025	HRFI
KO00532	Glycosaminoglycan biosynthesis - chondroitin sulfate / dermatan sulfate	−1.52	0.040	HRFI

^a^ *NES* normalized enriched score

^b^ *FDR* false discovery rate

Positive and negative NES indicate higher and lower expression in LRFI, respectively

### Validation of RNA-seq results

**Fig. 7:**
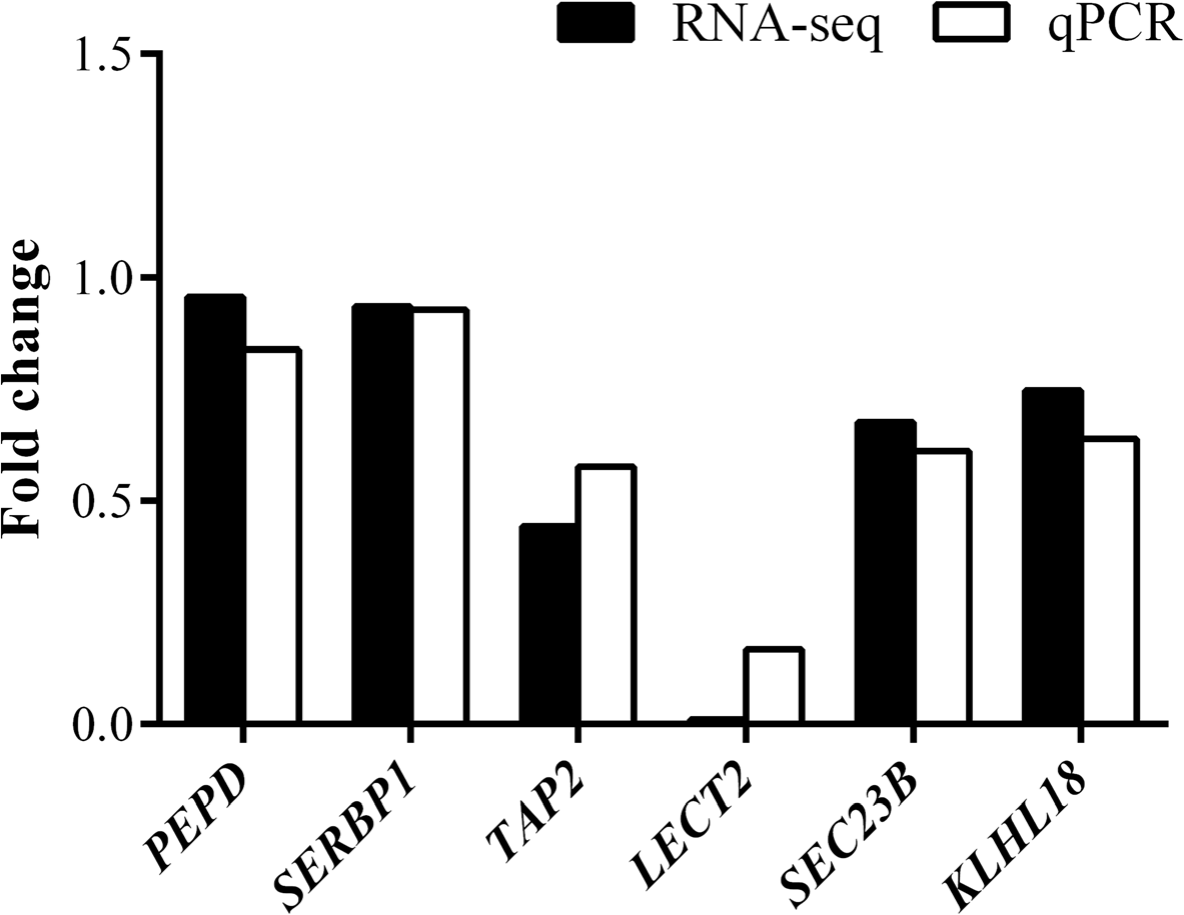
Validation of the differential expression genes in the breast muscle of the native chickens. *RNA-Seq* RNA Sequencing, *qPCR* quantitative real-time polymerase chain reaction, *PEPD* peptidase D, *SERBP1* SERPINE1 mRNA binding protein 1, *TAP2* transporter 2, ATP-binding cassette, sub-family B (MDR/TAP), *LECT2* leukocyte cell derived chemotaxin 2, *SEC23B* Sec23 homolog B, coat complex II component, *KLHL18* kelch like family member 18, *GAPDH* glyceraldehyde-3-phosphate dehydrogenase

To validate RNA-seq expression profiles, six genes were selected randomly from all differential expression genes. These genes are *PEPD* (peptidase D), *SERBP1* (SERPINE1 mRNA binding protein 1), *TAP2* (transporter 2, ATP-binding cassette, sub-family B), *LECT2* (leukocyte cell derived chemotaxin 2), *SEC23B* (Sec23 homolog B, coat complex II component), and *KLHL18* (kelch like family member 18). The samples of qPCR were same as samples utilized for RNA-seq. The qPCR analysis confirmed that the selected genes were differently expressed between the RFI groups, indicating that RNA-seq results were accurate and reproducible (Fig. 7).

## Discussion

In this study, the breast muscle transcriptome data were obtained from two groups of native chickens with extreme opposing RFI using high-throughput RNA-seq technology. The gene expression profile was deconstructed and understood by an integrated bioinformatics analysis. Firstly, the DEGs were identified from transcriptome data and analyzed by functional annotation. Secondly, an in-depth analysis of DEGs was performed by the integration of PPI network and module analysis. Meanwhile, the hub genes were identified through the analysis of key nodes in the PPI network. Finally, all expressed genes were ranked according to the strength of expression difference, and then a GSEA method was employed for functional enrichment between RFI groups. All bioinformatics analyses investigated the differences, associations, and enrichment of expressed genes from the above three different perspectives in order to further gain a comprehensive biological insight into the feed efficiency of native chickens.

### Functional annotation and biological interpretation of DEGs

Typical differential expression analysis of transcriptome data produces a list of hundreds of DEGs, requiring further analysis to construct a high-level overview of changes between the different compared groups [22]. In this study, a total of 349 known DEGs (24 up-regulated and 325 down-regulated) were identified from sequencing data. Ontology annotation of DEGs revealed several biological events related to immune system process, response to stimulus and T cell activation. ‘immune system process’ was the most significantly enriched GO term in the LRFI birds relative to the HRFI birds. All genes of this term were down-regulated in LRFI group. Moreover, a range of GO terms related to immune response, including ‘regulation of immune system process’, ‘regulation of immune response’, and ‘activation of immune response’, were found significantly enriched in LRFI groups. It was widely considered that immune response may increase maintenance requirements, and are prioritized overgrowth in terms of nutrient allocation [23]. Nutrients shifted away from growth toward the immune-related processes may reduce feed efficiency in animals during the immune response [24]. Moreover, a range of GO terms involved with the response to stimulus, such as ‘regulation of response to stimulus’, ‘response to stimulus’, and ‘positive regulation of immune response’, were found enriched in the LRFI group compared to the HRFI group in breast muscle. Similar to the genes of GO terms related to immune response, almost all genes of these GO terms were down-regulated in LRFI group. A previous study indicated that LRFI heifers respond differently to hepatic proinflammatory stimulus and may expend less energy toward combating systemic inflammation and redirecting nutrients toward growth and protein accretion [25]. Our finding indicated that genes related to immune response and response to stimulus may be important factors contributing to the difference in feed efficiency. In agreement, it was well documented that pigs with high feed efficiency showed lower susceptibility to oxidative stress during production compared to pigs with low feed efficiency, resulting in lower inflammatory responses and lower growth impairment [26]. Moreover, a previous study in pigs indicated that genetic selection for low RFI (high feed efficiency) resulted in less stress responsiveness [27].

We further analyzed the KEGG pathways of DEGs, including 8 significantly enriched pathways. These enriched pathways focus on immune response, response to cytokine, energy metabolism, and inflammatory response. Among these, the top 3 pathways, including ‘phagosome’, ‘cell adhesion molecules (CAMs)’ and ‘intestinal immune network for IgA production’, are associated with immune response and inflammatory response. 2 pathways, including ‘cytokine-cytokine receptor interaction’ and ‘Jak-STAT signaling pathway’, were involved in the response to cytokine. Cytokines are a group of proteins that are soluble in water and secreted by various cells primarily in response to stimulus and responsible for transmitting messages between cells. It was well documented that the over-production of pro-inflammatory cytokines may lead to damage to intestinal integrity and epithelial function and subsequently reduced feed efficiency [28]. These results indicated that pathways related to immune response and inflammatory response are associated with feed efficiency. Consistent with previous studies, it is well established that LRFI pigs have an up regulated basal colonic inflammatory state and a heightened response to a lipopolysaccharide (LPS) challenge compared with the HRFI pigs [29]. A similar finding suggested that compared with low feed efficiency pigs, the high feed efficiency pigs could induce a quicker and more effective hepatic response to inflammatory stimuli [24].

Interestingly, we found only genes (*ND1, ND2, ND3, ND4, ND4L, ND5, CYTB, COX1, COX2, COX3,* and *ATP6*) of ‘oxidative phosphorylation’ were up-regulated in LRFI group, while genes of other enriched pathways were down-regulated in LRFI group. Based on the figure of oxidative phosphorylation pathway (Fig. 2), the up-regulated genes of this pathway appear in major mitochondrial complexes, including complex I, complex III, complex IV, as well as ATP synthase (complex V). These mitochondrial complexes form the electron transport chain [30], which coupled the oxidative phosphorylation to produce energy in mitochondria [31]. Among these, *ND1, ND2, ND3, ND4, ND4L,* and *ND5* are the core subunits of the mitochondrial membrane respiratory chain NADH dehydrogenase (complex I), which is the largest mitochondrial complex and has the entry site of the NADH electron transfer chain [32]. Notably, *ND2*, *ND4*, and *CYTB* were top 10 up-regulated DEGs in the breast muscle of LRFI group compared to HRFI group (Table 2). *ND2* plays a key role in controlling the production of the mitochondrial reactive oxygen species (ROS), which can contribute to oxidative damage to mitochondrial structure and functions. It was reported that the missense substitution in the *ND2* was significantly associated with the production of ROS in Tibet chicken [33]. *ND4* protein is a hydrophobic inner membrane subunit of mitochondrial complex I and is thought to be involved in the proton translocation function of complex I [34]. Previous research indicated that the expression of *ND4* and *COX 2* was lower in the low feed efficiency broilers compared with high feed efficiency broilers [35]. *CYTB* is one of the 11 subunits of mitochondrial complex III, and is the key to maintain the function of complex III [36]. Mutations in *CYTB* might result in the functional failure of complex III, which could have a negative impact on complex I function [37]. Moreover, A previous study suggested that the presence of mtDNA polymorphisms, including *ND4, CYTB*, and *COX3*, affecting respiratory chain complexes I, III and IV, and were associated with altered ROS level [38]. The aforementioned results confirmed that LRFI chickens may have tighter control over ROS production compared with HRFI chickens through enhancing the expression of genes related to mitochondrial function. Coincidentally, it was established that low feed efficiency broilers produced higher amounts of ROS compared with high feed efficiency broilers [39]. Moreover, a previous study suggested that using poor hygiene conditions to activate mature fat cells isolated from different RFI pigs could lead to higher ROS production in HRFI pigs [26]. Hence, it can be inferred that *ND2*, *ND4*, and *CYTB* were key candidate genes affecting feed efficiency in native chickens.

### Integration of PPI network and module analysis

The alignment and mapping of PPI networks provide opportunities to further investigate the intrinsic relationship between DEGs through conserved pathways and protein complexes [40]. Analyzing PPI network is an important prerequisite for understanding the molecular basis for complex traits. In our study, the PPI network was constructed with DEGs, and then the top centrality hub genes were obtained using four centrality methods. Finally, we identified 4 hub genes, including *RAC2*, *VCAM1*, *CTSS*, and *TLR4*. *RAC2* was identified as one of the hub genes with the highest degree of connectivity. *RAC2* is a key signal transduction factor in inflammatory cells and plays a key role in the activation of the various NADPH oxidases (NOXes) family members, which play important role in the production of ROS through response to receptor agonists such as growth factors or inflammatory cytokines [41]. Moreover, a previous study suggested that *RAC2* deficiency inhibits the action of pro-inflammatory cytokines and chemokines [42]. In chickens, *RAC2* is involved in the production of ROS in phagosomes of chicken heterophils to kill pathogens [43]. *VCAM1* encode vascular cell adhesion molecule-1 and mainly expressed in endothelial cells during inflammation [44]. Dysfunctional endothelial cells express adhesion molecules and release *VCAM1*, thereby causing vascular inflammation [45], and this event appears to be mediated by increased ROS production [46]. *CTSS*, encoding for cathepsin S protein, is implicated in body weight regulation and the development of obesity [47]. It was reported that *CTSS* expression and cathepsin S in adipose tissue were induced by pro-inflammatory factors, such as TNF-α and IL-β [48]. *TLR4* is a member of toll-like receptors (TLRs) family, which recognize mainly microbial membrane components [49]. *TLR4* is also the only known member of the TLR family that engages all four toll-interleukin receptor (TIR) domains-containing adaptor proteins to participate in signaling inflammatory response [50]. A previous study indicated the elevated *TLR4* expression in skeletal muscle expression may lead to augmented inflammation and chronic disease risk observed with increased adiposity [51].

It is worth signaling that, the four hub genes were mainly expressed in inflammatory cells. Under normal circumstances, skeletal muscle is responsible for most insulin-stimulated glucose processing throughout the body. Existing evidence suggested that skeletal muscle myocytes can secrete large amounts of cytokines and other molecules and may become inflamed in obesity [52]. Moreover, skeletal muscle myocytes can express and secrete numerous cytokines such as *IL-6*, *IL-15*, and other molecules such as irisin and myonectin, whereas most adipokines are pro-inflammatory, regulated by obesity [53]. Furthermore, a previous study indicated that immune cells can also cause myocyte inflammation by secreting pro-inflammatory molecules for adverse regulatory effects on myocyte metabolism [54]. In summary, the four hub genes obtained from the PPI network were up-regulated in skeletal muscle of HRFI chickens and deeply involved in the production of ROS and inflammatory response. In this study, the four hub genes up-regulated in HRFI chickens, which indicated the HRFI chickens increased ROS production and inflammatory response. In agreement, a number of studies have suggested that low feed efficiency pigs showed higher inflammatory responses, growth impairment, and ROS production [26, 29]. Similarly, in the above DEGs enrichment analysis, our results indicated that the birds in the HRFI group up-regulated inflammation-related pathways, such as ‘phagosome’, ‘cell adhesion molecules (CAMs)’, and ‘cytokine-cytokine receptor interaction’, and down-regulated genes related to mitochondrial function.

In this study, the HRFI group consumed 8.8% more feed than the LRFI group. The overconsumption of food of HRFI chickens may lead to metabolic disorders and overload of the electron transport chain, which increased the production of ROS and resulting in cellular oxidative stress [55]. A previous study indicated that the generation of ROS level lead to numerous downstream effects, including triggering inflammatory cascades and increasing production of ROS [56]. In chickens, the blunted inflammatory response may reduce feed demand and stimulate faster muscle growth [57]. Hence, according to the aforementioned results, it could be hypothesized that overconsumption of food may increase the risk of overload of electron transport chain, which in turn leads to cellular oxidative stress and inflammatory response, resulting in increased feed demand and reduced feed efficiency in HRFI chickens.

To further analyzed the PPI network, we constructed three significant modules (Fig. 5). In the current study, the genes of module 1 were up-regulated in HRFI group and enriched in ‘phagosome’ and ‘cell adhesion molecules (CAMs)’ pathway. The seed node of module 1 is *IL16*, which is a polypeptide pro-inflammatory cytokine that plays a key role in most immune and inflammatory responses [58]. This result further confirmed the above surmise that HRFI chickens increased inflammatory response. Genes of module 2 were up-regulated in LRFI chickens and enriched in ‘oxidative phosphorylation’ and ‘metabolic pathways’. The seed node of module 2 is *ATP6*, which plays a crucial role in the proton channel of ATP synthase (complex V). A previous study indicated that the mutation of *ATP6* gene may make Tibetan chickens easier to convert energy and metabolize than Chinese native chickens [59]. This finding is consistent with the above results that LRFI chickens enhanced expression of genes related to mitochondrial function. Genes of module 3 are involved actin cytoskeleton, which implicated in the regulation of cell motility [60]. Thus, it can be speculated that the ‘phagosome’ and ‘cell adhesion molecules (CAMs)’, and ‘oxidative phosphorylation’ were key pathways affecting feed efficiency in native chickens.

### Gene set enrichment analysis

In the current study, we used GSEA method to convert the RNA-seq count data into biological interpretations. In this way, we do not rely on any arbitrarily predefined threshold to select ‘interesting’ genes or pathways for function analysis. And GSEA can accurately and reliably detect gene sets with biological meaningful [17]. In this study, based on the GO-based list, all higher expressed gene sets in LRFI group were mainly related to mitochondrial function. Among these, the ‘mitochondrial membrane part’ and ‘electron transport chain’ were significantly enriched gene sets (Fig. 6). These results indicated that LRFI chickens increased mitochondrial function, especially in function of electron transport chain. The results were consistent with the former function analysis of DEGs that LRFI chickens enhanced the expression of genes related to mitochondrial complexes, which form the electron transport chain (Fig. 2). ‘Negative regulation of cytokine-mediated signaling pathway’ and ‘negative regulation of response to cytokine stimulus’ were higher expressed in HRFI chickens. This result indicated that the aforementioned two pathways related to cytokine, including ‘cytokine-cytokine receptor interaction’ and ‘Jak-STAT signaling pathway’, should deserve more attention in further research.

Base on the KEGG-based list, we found that ‘citrate cycle (TCA cycle)’ was the most significantly enriched gene set, with higher expression in LRFI group compared with HRFI group. It was well known that the TCA cycle is the major common pathway for oxidation of carbohydrates, lipids, and some amino acids, and finally results in the production of large amounts of adenosine triphosphate (ATP) via oxidative phosphorylation [61]. This result indicated that LRFI chickens increased the expression of genes of the ‘citrate cycle (TCA cycle)’ pathway in skeletal muscle. It was well documented that mitochondria are involved in ATP synthesis through the TCA cycle and oxidative phosphorylation [62]. Based on the above analysis, we speculated that compared with the HRFI chickens, LRFI chickens may synthesize ATP more effectively by enhancing TCA cycle and oxidative phosphorylation in skeletal muscle. In agreement, a recent study suggested that high feed efficiency broilers enhanced expression of the energy production in breast muscle [63]. Moreover, a recent study in pigs suggested that compared with HRFI pigs, LRFI pigs might be more efficient in energy utilization during skeletal muscle growth [64]. Furthermore, a previous study indicated that high feed efficiency pigs can use nutrients more effectively to promote growth than low feed efficiency pigs [24]. Collectively, our results of GSEA indicated that LRFI chickens had higher expression of genes related to mitochondrial function compared with HRFI chickens, and the ‘citrate cycle (TCA cycle)’ may be a key pathway to influence the feed efficiency of native chickens.

## Conclusions

In summary, we performed RNA-seq analysis on breast muscle derived from native chickens with extreme opposing RFI. Enrichment and interaction analysis of DEGs and GSEA method were employed for further analysis to construct a high-level overview of changes between the different RFI groups. Our results indicated that *ND2*, *ND4*, *CYTB*, *RAC2*, *VCAM1*, *CTSS* and *TLR4* were key genes affecting feed efficiency of native chickens, and they may influence feed efficiency through deep involvement in ROS production and inflammatory response. Function analysis of DEGs and GSEA analysis suggested that genes related to immune response, mitochondrial function, response to stimulus, and inflammatory response are associated with feed efficiency. Moreover, the ‘phagosome’, ‘cell adhesion molecules (CAMs)’, ‘citrate cycle (TCA cycle)’ and ‘oxidative phosphorylation’ were key pathways contributing to the difference in feed efficiency. Among these, Genes and pathways related to inflammatory response and immune response were up-regulated in HRFI chickens, while genes and pathways related to mitochondrial function were up-regulated in LRFI chickens. Our study indicated that HRFI chickens may face more oxidative stress and the consequent increased inflammatory response, while LRFI chickens may synthesize ATP more efficiently and control ROS production more strictly by enhancing the mitochondrial function in skeletal muscle. The interaction between inflammatory response and mitochondrial function in skeletal muscle needs further investigation to understanding the underlying molecular mechanisms affecting the feed efficiency of native chickens.

## Methods

### Birds and RFI calculation

A pedigreed chicken population was established from a random breed population, 200 males mated with 1000 females obtain 4500 chickens in one hatch. All birds used in the current study were provided by Qingyang Pingyun Poultry Conservation and Breeding, Co. Ltd. After hatch, a total of 2500 male Wannan Yellow chicken were selected and raised as experimental populations. At 56 day of age, a total of 1008 chickens with similar body weight were selected and transferred to individual cage, each cage measuring 45 cm × 35 cm × 50 cm. All chickens had access to water ad libitum. All chickens were fed the same diet throughout the experiment period, which provided by Qingyang Pingyun Poultry Conservation and Breeding, Co. Ltd.

The feed intake and ADFI were measured at 56–98 d of age. The BW56 and BW98 were recorded to calculate the MBW^0.75^, BWG, and ADG. FCR was calculated by FI and BWG, RFI is calculated as difference between the actual and expected FI using the model as follows [20]:

$$ \mathrm{RFI}=\mathrm{ADFI}-\left({b}_0+{b}_1\mathrm{ADG}+{b}_2{\mathrm{MBW}}^{0.75}\right), $$

where ADFI, ADG, and MBW^0.75^ are the average daily feed intake, average daily body weight gain, and metabolic body weight, respectively. Additionally, *b*_*0*_ is the regression intercept, *b*_*1*_ is the partial regression coefficient of ADFI on ADG, and *b*_*2*_ is the partial regression coefficient of ADFI on metabolic weight. The RFI values were calculated using the regression procedure of SAS (version 9.4, SAS Inst. Inc., Cary, NC). After excluding outlier data (total 1.5%), all chickens were ranked according to the RFI value. 30 highest RFI (HRFI) chickens and 30 lowest RFI (LRFI) chickens were selected as HRFI and LRFI group.

All animal performance data showed in the table are expressed as least square means ± standard error of the mean (SEM). Student’s *t*-test was used to analyze the feed efficiency difference between HRFI and LRFI groups. The probability value was *P* < 0.05, indicating statistical significance.

### RNA extraction and sequencing

At the age of 98 days, 5 birds were randomly selected from HRFI group and LRFI group, respectively. All birds were manually killed by cervical dislocation. The pectoralis major was immediately collected and stored in liquid nitrogen and subsequently transferred to the laboratory and stored at − 80 °C for further use (RNA sequencing). Total RNA was extracted from the pectoralis major (100 mg) using TRIzol reagent (Invitrogen, Carlsbad, CA, USA) based on the manufacturer’s instructions. RNA quality was determined by measuring the absorbance at 260, 280 and 230 nm using NanoDrop 2000 (Thermo Fisher Scientific). The reference 260/280 ratio and 260/230 ratio for the RNA sample were 1.8 to 2.0 and 1.8 to 2.2, respectively. The integrity number was tested by Agilent 2100 Bioanalyzer (Agilent, Santa Clara, CA, USA, 2009). Only RNA integrity number equal to or higher than 7.0 was RNA used for the next analysis.

After total RNA was extracted and checked, all samples were sent to Genedenovo Biotechnology Co., Ltd. (Guangzhou, China) for cDNA library construction. All samples were sequenced using the Illumina HiSeq 4000 platform (Illumina, San Diego, California, USA).

### RNA-seq data analysis

Before read alignment, the quality control of raw sequence reads was performed using the FastQC program (version 11.5, http://www.bioinformatics.babraham.ac.uk/projects/fastqc/) and nucleotide calls with a quality score of 30 or higher were considered high quality clean reads. Adapters and low-quality reads were trimmed using the Cutadapt (1.14) such that the average base quality was greater than 20.

After trimming, the processed reads were then aligned to the chicken reference genome GRCg6a (GCA_000002315.5) using the alignment program Tophat2 (version 2.1.1, http://ccb.jhu.edu/software/tophat/index.shtml). The reference genome and annotated file were obtained from the Ensembl database (http://asia.ensembl.org/Gallus_gallus/Info/Index). After aligned with the reference genome, unmapped reads were then re-aligned with Bowtie2, the enriched unmapped reads were split into smaller segments which were then used to find potential splice sites. Then, a reference annotation-based transcript assembly for each sample was performed using the Cufflinks (version 2,2,1). The fragments per kilobase of exon per million reads (FPKM) value was used to quantify the gene expression levels. In addition, all assembled transcripts of all samples were merged to improve the overall quality of assembly by merging new and mapped transcripts into a single assembly and deleting artificial structures.

### Identification of differently expressed genes (DEGs) and function annotation analysis

DEGs were identified using Cuffdiff (version 2.2.1), here, only identified transcripts with a fold change > 2 or < 0.5, and a false discovery rate (FDR) < 0.05 were used for subsequent analysis.

To identify the biological function related to the DEGs, the Kyoto Encyclopedia of Genes and Genomes (KEGG) pathways and Gene Ontology (GO) terms (CC, Cellular Component, MF, Molecular Function, BP, Biological Process) were investigated using the Database for Annotation, Visualization and Integrated Discovery (DAVID) (version 6.8, https://david.ncifcrf.gov/) [65]. The GO terms and KEGG pathways with Benjamini-Hochberg (B-H) *P* value < 0.5 were considered to be statistically significant enrichment.

### Protein-protein interaction (PPI) network construction and modules selection

The Search Tool for the Retrieval of Interacting Genes (STRING) database was used to obtain PPI data. Mapping DEGs to STRING to evaluate the interactive relationship, with a confidence score > 0.9 defined as significant. PPI network of DEGs was visualized by Cytoscape (http://cytoscape.org/), which is an open source software for visualizing complex networks and integrating them with any type of attribute data. The CytoHubba application in Cytoscape was performed to analyze the hub genes through four centrality methods, including Degree, EPC, EcCentricity, and MNC [66]. The Molecular Complex Detection (MCODE) [67] application in Cytoscape was used to screen the modules of the PPI network. The criteria setting of MCODE is: degree cutoff = 2, node score cutoff = 0.2, k-core = 2, maximum depth = 100. Moreover, the function and pathway enrichment analysis was performed for genes in the modules.

### Gene set enrichment analysis (GSEA)

All expressed genes, regardless of whether they were differentially expressed in either case, were used for GSEA analysis. Gene set analysis was analyzed by GSEA software (http://software.broadinstitute.org/gsea/index.jsp) based on C5, CC, C5.BP, C5.MP, and C2.CP:KEGG gene set collections (MSigDB v7.0, broad institute, Cambridge, MA, USA) [68]. GSEA first ranked all expressed genes according to the significance of differential gene expression between the HRFI and LRFI groups. The enrichment score for each gene set is then calculated using the entire ranked list, which reflects how the genes for each set are distributed in the ranked list. Normalized enriched score (NES) was determined for each gene set. The significant enrichment of gene set was selected based on the absolute values of NES > 1, nominal *P*-value of NES ≤ 0.05, and false discovery rate (FDR) ≤ 0.05 [69].

### Validation of RNA-seq through quantitative real-time PCR (qPCR)

Following cDNA synthesis from 1 μg of total RNA and in presence of random primers (Promega, Mannheim, Germany), these primers were designed using Primer 5.0 software and synthesized by the Nanjing Tsingke biological technology Co. Ltd. (Nanjing, China). The primer sequences are provided in (Additional file 6: Table S6). First-strand complementary DNA (cDNA) was synthesized using one-step gDNA Removal and cDNA Synthesis SuperMix (TransGen Biotech Co., Ltd., Beijing, China) according to the manufacturer’s instructions. qPCR was carried out on a 7500 Real-Time PCR apparatus (Applied Biosystems, Warrington, UK) using the SYBR Green Master Mix (Biomiga, San Diego, CA, USA). The efficiency of the quantitative PCR reaction was verified by creating a standard curve from fivefold serial dilutions of cDNA. PCR reactions were carried out in a final volume of 20.0 μL, which contained 1.0 μL of 1000 ng cDNA, 1.0 μL of 10 μM forward and reverse primer mix, 10.0 μL 2 × SYBR green Master Mix, 8.0 μL RNase-free ddH_2_O. Samples were run in triplicate. The quantitative PCR program was at 95.0 °C for 5 min, 40 cycles of 95.0 °C for 15 s and 60.0 °C for 1.0 min, followed by a melting curve program was 1 cycle of 95.0 °C for 15 s, 60.0 °C for 1.0 min, 95.0 °C for 15 s, 60.0 °C for 15 s. The qPCR results were detected using a dissociation curve analysis and gel electrophoresis. CT-method was utilized to quantify the changes in the gene expression, whereas *GAPDH* served as a housekeeping for normalization. Relative gene expression was calculated using 2^-ΔΔCT^ method according to a previous study [70].

## Supplementary information

**Additional file 1: Table S1.** Sequencing data filtering and comparison of reference genomes.

**Additional file 2: Table S2.** All differentially expressed genes (DEGs) between HRFI and LRFI groups.

**Additional file 3: Table S3.** All enriched GO terms of differentially expressed genes in breast muscle between the HRFI and LRFI groups

**Additional file 4: Table S4.** GO and KEGG analysis of genes in each module

**Additional file 5: Table S5.** An entire result of Gene set enrichment analysis (GSEA) between HRFI and LRFI birds

**Additional file 6: Table S6.** Forward and reverse primers for RNA-seq validation through qPCR

## Data Availability

The datasets used and/or analyzed during the current study available from the corresponding author on reasonable request. The RNA sequencing data generated during the current study has been uploaded to the NCBI SRA database [https://www.ncbi.nlm.nih.gov/sra]. The accession numbers of RNA-seq data of the ten samples are SRR10343464, SRR10343463, SRR10343462, SRR10343461, SRR10343460, SRR10343469, SRR10343468, SRR10343467, SRR10343466, SRR10343465, respectively.

## Abbreviations

RFI: Residual feed intake
RNA-seq: RNA sequencing
DEGs: Differentially expressed genes
GSEA: Gene Set Enrichment Analysis
BW56: Initial body weight
BW98: Final body weight
ADFI: Average daily feed intake
ADG: Average daily body weight gain
MBW^0.75^: Metabolic body weight
FCR: Feed conversion ratio
HRFI: Highest RFI
LRFI: Lowest RFI
SEM: Standard error of the mean
KEGG: Kyoto Encyclopedia of Genes and Genomes
GO: Gene ontology
DAVID: Database for Annotation, Visualization and Integrated Discovery
B-H: Benjamini–Hochberg method
STRING: The Search Tool for the Retrieval of Interacting Genes
PPI: Protein-protein interaction
NES: Normalized enriched score
FDR: False discovery rate
qPCR: Quantitative real-time PCR
log2FC: Log2 (fold change)
ROS: Reactive oxygen species
NOXes: NADPH oxidases
TLRs: Toll-like receptors
TIR: Toll-interleukin receptor
