# Supplementary material for: Identification of key genes and pathways associated with feed efficiency of native chickens based on transcriptome data via bioinformatics analysis
Source: BMC Genomics. 2020 Apr 9;21:292. doi: 10.1186/s12864-020-6713-y (PMC7146967; doi:10.1186/s12864-020-6713-y)
Supplement: Supplementary file 1 — Additional file 1: Table S1. Sequencing data filtering and comparison of reference genomes. [file 12864_2020_6713_MOESM1_ESM.pdf]

Table S1 Sequencing data filtering and comparison of reference genomes

Sample	Raw reads	High quality raw reads	Trimmed reads	Mapped reads	Mapping ratio
HRFI_9	79841656	78860208	77313274	64725580	83.72%
HRFI_11	79777378	78752858	77656894	65254902	84.03%
HRFI_13	91699444	90599596	88802990	73817210	83.12%
HRFI_15	94766182	93728314	92213654	77449619	83.99%
HRFI_21	85869118	84857816	82966998	69676560	83.98%
LRFI_9	77420606	76494894	75124186	61969884	82.49%
LRFI_10	88572774	87480298	80586384	66362319	82.35%
LRFI_14	86841400	85857868	80603060	67530066	83.78%
LRFI_19	81857788	80887906	79321372	66296801	83.58%
LRFI_27	92552470	91011846	85925220	68275443	79.46%
