# Supplementary material for: Identification of key genes and pathways associated with feed efficiency of native chickens based on transcriptome data via bioinformatics analysis
Source: BMC Genomics. 2020 Apr 9;21:292. doi: 10.1186/s12864-020-6713-y (PMC7146967; doi:10.1186/s12864-020-6713-y)
Supplement: Supplementary file 6 — Additional file 6: Table S6. Forward and reverse primers for RNA-seq validation through qPCR [file 12864_2020_6713_MOESM6_ESM.pdf]

Table S6 Forward and reverse primers for RNA-seq validation through qPCR

Gene symbol	GenBank nucleotide accession	Primer sequence[a] (5'→3')	Amplicon length (bp)
PEPD	NM_001079717.1	F: ACTGGACTTTCGGAGTAACGG	167
		R: GTGGACCTCATCCACAGCAT	
SERBP1	NM_001031293.1	F: CGGCAGTATCGGAGCAGGAT	120
		R: GGGTCGGACTCGTCATCAAA	
TAP2	NM_001099357.1	F: TGGGCTTCATCTCTGCACTG	138
		R: CGTCGAGGATAAGGACGGTG	
LECT2	NM_205478.2	F: GGGGATCAGGCTTCTGTGTC	157
		R: TCTGAGCGATCGCAGTTCTC	
SEC23B	NM_001006179.1	F: GGAGCTATTGGACCGTGTGT	115
		R: TTGTGCAGGGATCCAAACCG	
KLHL18	NM_001030960.2	F: CTGCTGCCACAAATGCAGAG	156
		R: GCTGCTGAGTTAAGTCCTCCA	
GAPDH	NM_204305.1	F: GGTGGCCATCAATGATCCCT	105
		R: CCGTTCTCAGCCTTGACAGT	

[a] F: forward primer; R: reverse primer.
